# Characterization of *Chromobacterium violaceum* pigment through a hyperspectral imaging system

**DOI:** 10.1186/2191-0855-4-4

**Published:** 2014-01-13

**Authors:** Maria J Gallardo, Juan P Staforelli, Pablo Meza, Ignacio Bordeu, Sergio Torres

**Affiliations:** 1 Center for Optics and Photonics, Universidad de Concepción, Concepción, Chile; 2 Departamento de Física, Universidad de Concepción, Concepción, Chile; 3 Departamento de Ingeniería Eléctrica, Universidad de La Frontera, Temuco, Chile; 4 Departamento de Ingeniería Eléctrica, Universidad de Concepción, Concepción, Chile

**Keywords:** Chromobacterium, Hyperspectral systems, Real time sampling, Pigment production

## Abstract

In this paper, a comprehensive spatio-spectral and temporal analysis for *Chromobacterium violaceum* colonies is reported. A hyperspectral imaging (HSI) system is used to recover the spectral signatures of pigment production in a non-homogeneous media with high spectral resolution and high sensitivity in *vivo*, without destructing the sample. This non-contact sensing technique opens avenues to study the temporal growing of a specific section in the bacterial colony. Further, from a 580 [nm] and 764 [nm] spatio-spectral time series, a wild-type and mutant *Chromobacterium violaceum* strains are characterized. Such study provides quantitative information about kinetic parameters of pigment production and bacterial growing.

## Introduction

For decades, natural pigments have been extensively used in various fields of everyday life such as food production, textile industries, paper production, agricultural practices and researches, water science and technology (Arad and Yaron 1992; Sirimanne et al. 2006). Natural pigments not only have the capacity to increase the marketability of products, they also display advantageous biological activities as antioxidants and anticancer agents (Stahl and Sies 2005; Kong et al. 2010). Several intensely colored compounds have been isolated from certain microorganisms and from various environmental sources (Ahmad et al. 2012). An example is the violacein pigment, a purple-colored dye produced by one of the strains of *Chromobacterium violaceum*, which is an indole derivative (Duran and Menck 2001) with antitumoral, antibacterial, antiulcerogenic, antileishmanial, and antiviral activities. (Leon et al. 2001; Duran et al. 2007).

The detection of bacterial components like antibiotics, enzymes and secondary metabolites like pigments, is important in biotechnological industries (Steele and Stowers 1991). Traditional methods are effective but can take hours or days to realize them. Besides, they are destructive as well as time consuming. On the other hand, molecular detection based techniques for bacteria identification are rapid, specific and sensitive. However, most are technically complicated and costly, and require well-trained specialists (Steele and Stowers 1991; Lazcka et al. 2007; Velusamy et al. 2010). Now, molecular spectroscopy provides less invasive or even non-destructive methods that have been proven well-suited to investigate the chemical composition of biological systems (Cen and He 2007).

Hyperspectral imaging (HSI) is an emerging technique that assimilates spectroscopy and imaging to provide both spectral and spatial information of a biological sample (Plaza et al. 2009). Further, the potential that HSIs have for in vivo optical diagnostics have being exploited due to the non-invasive feature and the massive spatio-spectral information collected by the system (Vo-Dinh 2004; Pisani et al. 2013). In this sense, HSI systems record, for each spatial location being imaged, a set of hundreds of high spectral resolution images that jointly conform the spectrum of the biological sample. Such collections of images is known as hypercube (Borengasser et al. 2008), from which the pertinent qualitative and quantitative information is obtained (Sankaran et al. 2010; Siripatrawan et al. 2011). Different optical techniques can be applied to generate the hypercube from biological samples, some of them are based on tunable optical filters (Gat 2000), imaging spectrometers (Jun et al. 2009), and coded hyperspectral imaging (Studer et al. 2012), among other. In particular, push-broom hyperspectral cameras (PBHCs) are imaging devices that carried out the spectral decomposition by means of an optical process (Kim et al. 2010). Further, PBHCs employ a scanning procedure to record spectral information of one spatial line at a time, with the ability to scan multiple batches of samples simultaneously by moving across the process line. Also, the spatial information is important for monitoring the sample as it can be used to extract its chemical mapping at different spatial points (Zavattini et al. 2003; Singh et al. 2010). In this regard, we can use HSI system to measure the spectral signature of chemical components, like bacterial pigments, inside the cells without altering the sample and its measurements even in a heterogeneous medium where spectrophotometric signal is not resolutive.

In this work, a spectral characterization of *Chromobacterium violaceum* pigment through a HSI is performed from the visible spectrum to part of the near infrared spectrum (400 nm to 1000 [nm] with 1,04 [nm] of spectral resolution). It is well known that a pigment changes the color of the reflected light as a result of selective color absorption. In this sense, the pigment is quantified associating its signal to the maximal optical absorption centered at 580 [nm] (Gerhardt, 1970; Koch, 1994). With HSI it is now possible to resolve signals and quantify the bacterial pigment with a remote, non-invasive and real-time procedure comparatively to a spectrophotometric measurement. Finally, a series of spectral signatures masking relevant information that can serve as an indicator of a specific bio-processes is obtained, *i.e.* pigment production and bacterial growing rate. In this case, despite using a small number of spectral bands, these are selected as a function of the amount of spectro-temporal information associated with the pigmentation process. For a different process, the evaluation of the spectral information can determine a larger number of spectral bands required for characterization. Hence, the amount of useful spectral information is directly related to the process being measured.

## Materials and methods

### Bacterial cultures

The *C. violaceum* wild-type strain ATCC 31532 and *C. violaceum* CV026 (mini-Tn5 mutant of ATCC 31532) (McClean et al. 1997) are cultured in Luria Bertani (LB) broth (10*%* peptone, 5*%* yeast extract, 5*%* NaCl, per 1 *L* distilled water) (Sambrook et al. 1989). Solid bacterial medium is made by the addition of agar at a final concentration of 15 *g*/*L*.

### Spectrophotometric measurement

The bacterial population and the pigment production is estimated by measuring absorbance of the culture using a spectro-photometer (UV-1700 UV-VIS Spectro-photometer) at different wavelengths (350-700 [nm]). Measurements are taken each one hour during lag phase and every 30 minutes in the exponential phase. For each measurement an aliquot of liquid culture is taken and the absorbance is determined every 50 [nm] (between 350 and 700 [nm]).

### Spot plating and incubation

**Figure 1:**
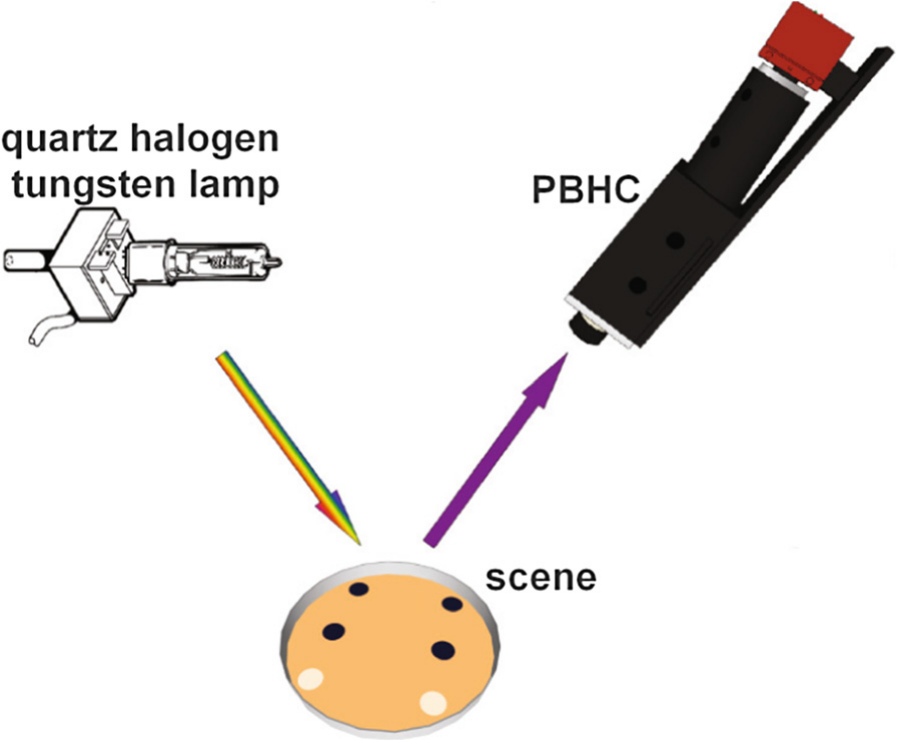
**Spot plating and simplified reflection based measurement scheme.** *Chromobacterium violaceum* wild-type strain ATCC 31532 (purple spots) and *C. violaceum* CV026 (white spots) are inoculated on a petri dish in LB agar. The plate is at uniform room temperature along the experiment.

The initial liquid bacterial culture is grown overnight at 30°C in a thermo-regulated bath. Agar LB plates are inoculated with 20 *μ*L spots at pre-determined and well-spaced locations on the agar surface. We separated the wild-type strain from the mutant strain to avoid contamination. Figure 1 shows a scheme of the imaged spot locations. The plate is incubated at room temperature in the camera stage for 24 hours. Three measurements are made, each one with four replicates of the wild-type strain and two replicates of the mutant strain.

### Experimental Setup

**Figure 2:**
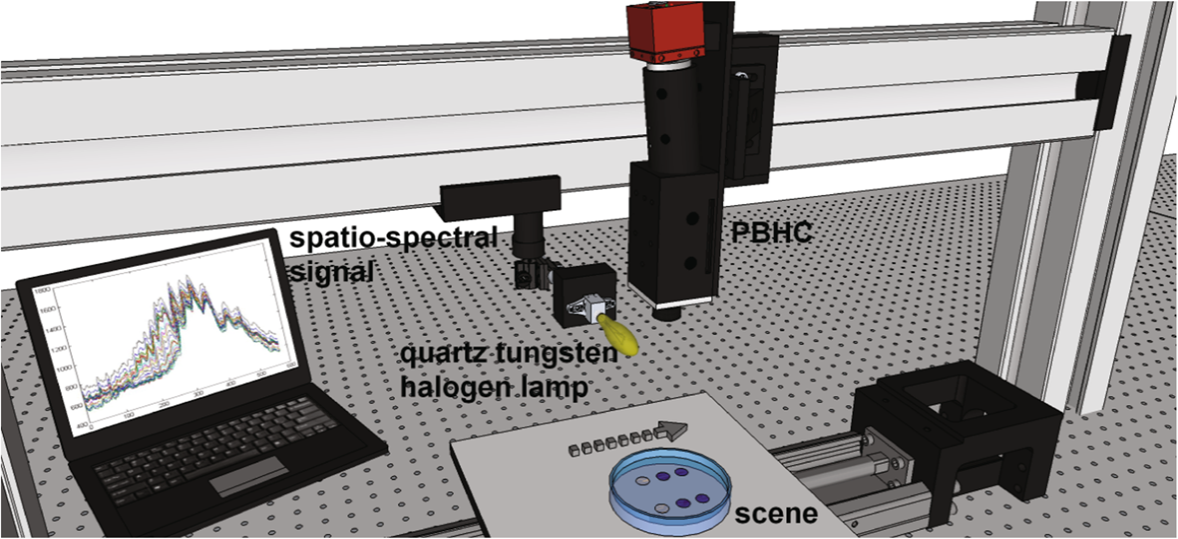
**Laboratory prototype to retrieve the spatio-spectral image.** The aluminum arc holds the illumination and camera system. The target scene lies on a movable base where the arrow indicates scan direction. Imaging is acquired during the scan process and data analysis is post-processed.

In order to acquire image sequences with high spectral resolution, The set-up illustrated in Figure 2 is constructed, where the key component is the PBHC. Such a camera can be described, in brief, as an optoelectronic imaging system composed of four sub-systems: the optics, the spectrograph, the sensor, and the readout electronics (Borengasser et al. 2008). Unlike broadband images, in PBHC the spatial information is mapped onto one axis of the focal plane array (FPA), while the spectral information is mapped onto the second axis. Therefore, a biological sample (now target scene) must be scanned one line at a time by displacing the camera in an orthogonal trajectory with respect to the axis used to encode the spectral information.

The HSI system employed in this work is the Photonfocus Hurricane 40 V10E PBHC. This camera is based on a CMOS FPA of 1024 × 1024 photo-detectors. Further, the input radiance is decomposed and converted into several spatio-spectral images through an optical process that allows us to obtain spectral bands between 400-1000 [nm], with a spectral resolution of 1.04 [nm]. Also, due to the nature of the acquisition process of PBHC, a mobile platform has been constructed to simulate and synchronize the scanning procedure.

Furthermore, to compensate for any noise source degrading the hyperspectral data, it is required to perform calibration measurements. To this end, we have employed a quartz tungsten halogen (QTH) calibration lamp, which guarantees an uniform and continuous spectral illumination between 200-2500 [nm]. Also, a Spectralon SRT- 99-120 is used as a diffuse reflectance target that ensures a reflectance of 99% between 250-2000 [nm].

As shown in Figure 2, the diffuse target lies on a petri dish holder to increase the reflectivity of the semi-transparent agar and colonies. Further, during the scanning process, each line of the target scene acquired receives the same angle of illumination, then we can ensure that all spatial points were illuminated under the same conditions. The sample measurement scheme is displayed in Figure 1, where the input radiance is generated by the light reflected from the agar. Finally, the dimensions of the hypercubes produced after scanning the whole target scene are 1024 spatial pixels, 574 spectral bands, and 1000 temporal lines.

### Spectral reflectance recovery

In order to retrieve the spectral reflectance of biological samples, several stages have to be performed. First, the sequence starts with the scene scanning due to the nature of the of the PBHC acquisition process. Second, the input radiance of a spatial line is decomposed and sampled into *P* spatial pixels and *Q* spectral bands, taking *S* line samples at different times. Hence, the entire target scene is mapped into a hypercube of dimensions *P* × *Q* × *S*. Third, the hyperspectral data must be calibrated to compensate for any degrading effect produced during the signal transduction. To do so, we have mathematically represented the hyperspectral response by the following first-order model:

(1) $$Y(i,\lambda_{j},k)=r(\lambda_{j})a(i,j)R(i,\lambda_{j},k)+b(i,j)+V(i,j,k),$$

where the suffixes *i*, *λ*_*j*_, and *k* denote, respectively, the spatial location at the scene, the spectral band, and the temporal sample, where *i* = 1,…,*P*, *j* = 1,…,*Q*^′^, and *k* = 1,…,*S*. The variable *R*(*i*,*λ*_*j*_,*k*) represents the input reflectance collected at the *λ*_*j*_th spectral band by the *ij*th photo-detector at the *k*th sample time. The terms *a*(*i*,*j*) and *b*(*i*,*j*) represent, respectively, all the multiplicative and additive noise sources corrupting the output of a PBHC. The additive term *V*(*i*,*j*,*k*) is known as the temporal white noise associated with the readout electronic for the *ij*th photo-detector. However, in most of the cameras the white noise *V*(*i*,*j*,*k*) is negligible compared to the term *b*(*i*,*j*), so it can be disregarded. Finally, the parameter *r*(*λ*_*j*_) represents the system spectral response, *i.e.* the slit, the diffraction grid response and the spectral responsivity of the detector material.

**Figure 3:**
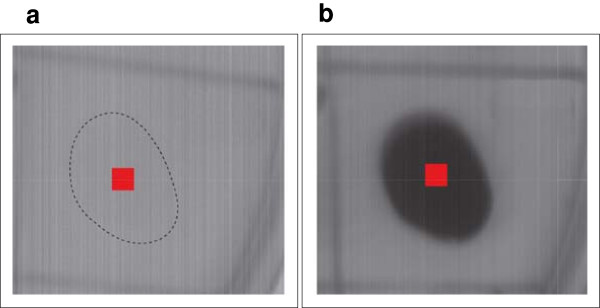
**Selected region of interest in the reasembled spectral image at 580 [nm] at a)** ***t*** **= 0 hrs and b)** ***t*** **= 37 hrs.**

In general, the additive noise term *b*(*i*,*j*) is treated as a baseline value produced by different noise sources, *i.e.*, dark current, non-uniformity and amplifiers noise. Such baseline can be estimated through the measurement of a dark reference. This is, considering that the spectral decomposition is carried out by an optical process, blocking the camera input prohibits the decomposition process, hence, *R*(*i*,*λ*_*j*_,*k*) = 0. Further, both multiplicative terms, *r*(*λ*_*j*_) and *a*(*i*,*j*) can be estimated by means of a white reference, which is obtained by illuminating a diffuse target with the QTH calibration lamp. Consequently, the following formula is used to compensate and retrieve the spectral reflectance:

(2) $$R(i,\lambda_{j},k)=\frac{Y(i,\lambda_{j},k)-\hat{b}(i,j)}{Y_{r}(i,\lambda_{j},k)-\hat{b}(i,j)},$$

where *Y*_*r*_(*i*,*λ*_*j*_,*k*) is the white reference value and $\hat{b}(i,j)$ is the estimated baseline obtained when measuring the dark reference. Recalling Eq. 1, by subtracting $\hat{b}(i,j)$ in Eq. 2, the baseline value is compensated. Further, the normalization between the measured sample and the white reference compensates for the system spectral response and the term *a*(*i*,*j*). With the spectral reflectance at hand, the absorbance values *A*(*i*,*λ*_*j*_,*k*) are retrieved and the maximal and minimal pigment absorption bands are selected, that in our case are 580 [nm] and 764 [nm], respectively. Therefore, to analyze the spectral behavior of the samples taken at different times, first it is required to reassemble the spatial information from the hypercube. Then, a region of interest region of interest (RI) is located on each sample, covering a spatial region equal to 21 by 21 pixels, as shown in Figure 3.

**Figure 4:**
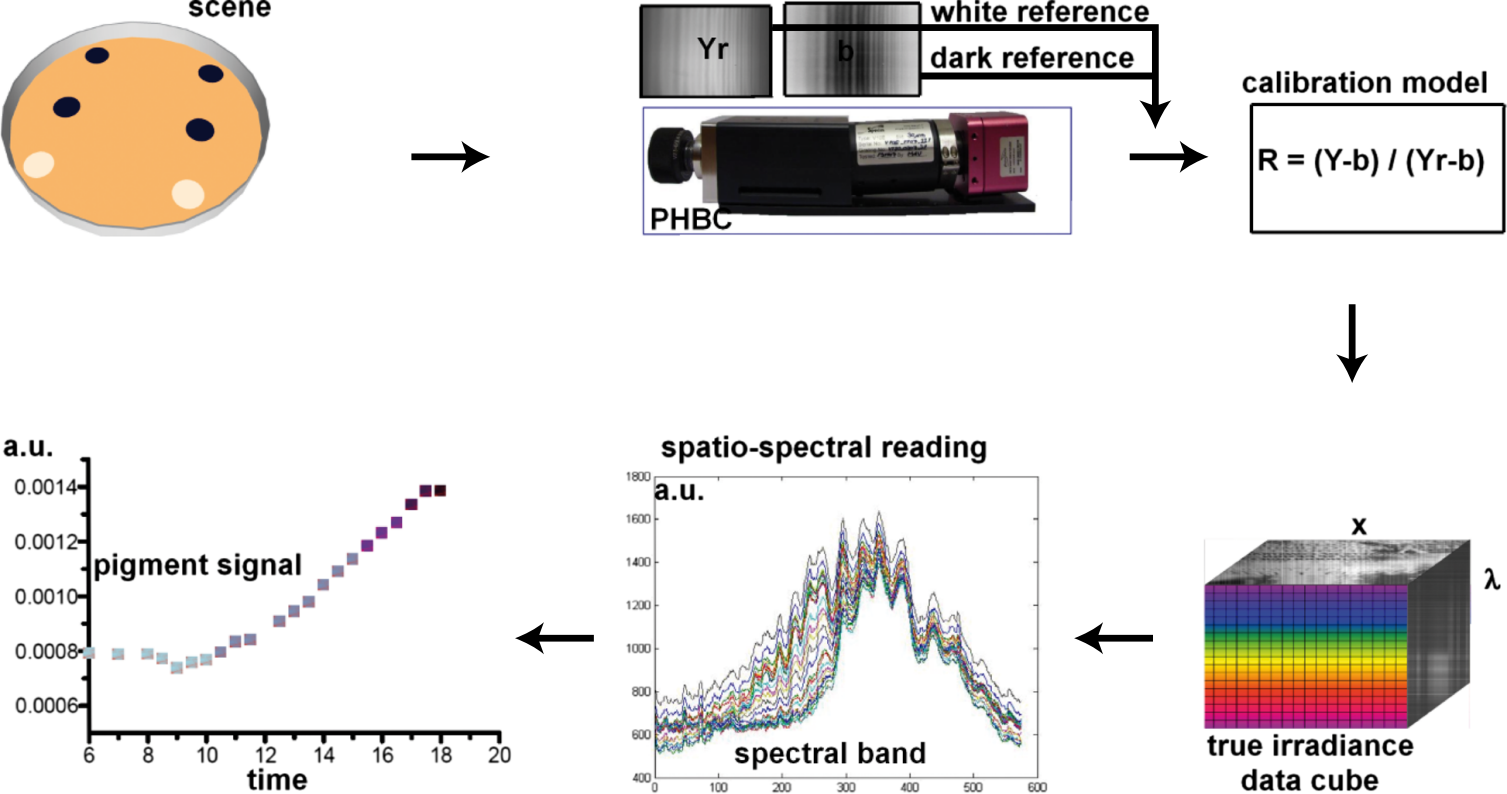
**Block diagram of the reflectance recovery process.** The sequence start with the scanning of the target scene. Data are then post-processed, here the calibration model compensates for the system spectral response which gives the spatio-spectral data cube. Then, the spatio-spectral reading identifies the specific wavelength of maximum absorbance that allows to characterize the pigment intensity change. Finally, the pigment signal is plotted as a function of time. Here, each point is a scanning sequence at that time. The scanning frequency is of the order of hours for the lag and stationary phase and half an hour for the exponential phase.

Each absorbance value enclosed by the RI is averaged to minimize any small variation produced by sensor noises or by simple irregularities. Thus, the sample averaged absorbance is calculated as follows:

(3) $$\bar{A}(i_{0},\lambda_{j},k_{0})=\overset{i_{0}+10}{\underset{n=i_{0}-10}{\sum }}\overset{k_{0}+10}{\underset{m=k_{0}-10}{\sum }}A(n,\lambda_{j},m)$$

where (*i*_0_,*k*_0_) is the RI center. It should be noted that since each pixel represents an absorbance value, selecting random measurement points is also a valid alternative. Finally, to summarize the methodology, a block diagram explaining each stage of the reflectance recovery process is presented in Figure 4.

## Results

**Figure 5:**
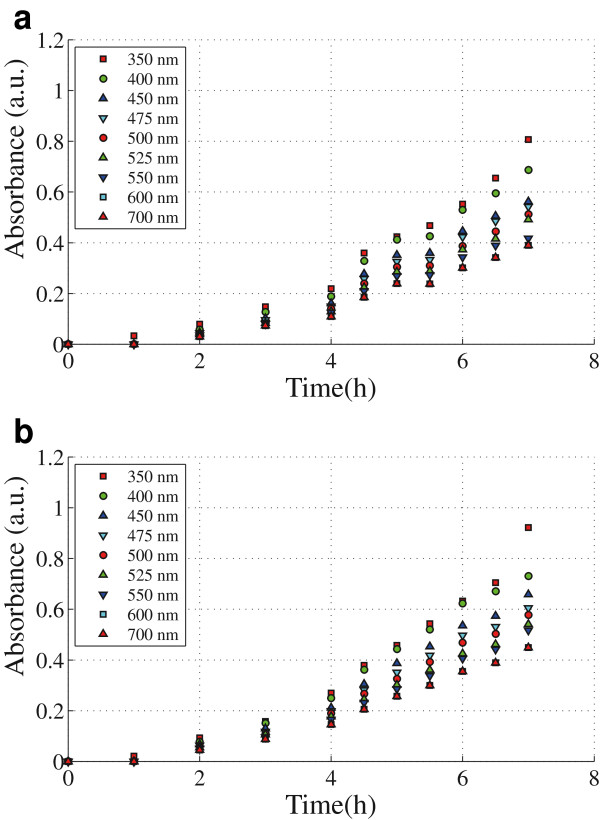
**Spectrophotometric measurement of both** ***C. violaceum* wild-type strain (a) and** ***C. violaceum* CV026 strain (b) in liquid culture.** Data shows the absorbance intensity signal versus time in the visible spectrum.

One of the advantages of using HSI systems is to recover the spectral signatures of pigment production in turbid media with high spectral resolution and sensitivity, in contrast to optical absorption based instruments. The traditional spectrophotometer procedure disposes the sample inside small quartz containers. Here, colored laser beams creates the observed absorption spectrum. When the sample is a complex mixture of bacterial cell components and pigment, the observed spectrum is indistinguishable for a clear identification, *e.g.*, between the *C. violaceum* wild-type strain and *C. violaceum* CV026 strain as shown in Figure 5.

**Figure 6:**
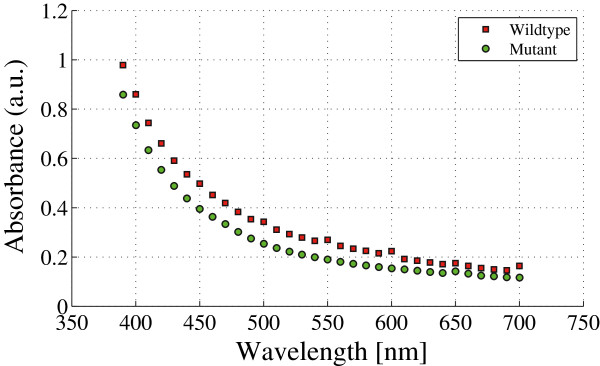
**Spectrophotometric measurement of both** ***C. violaceum* wild-type strain and** ***C. violaceum* CV026 strain in liquid culture.** Data shows the absorbance intensity signal versus wavelength. Data is taken during the stationary phase of growing, seven hours after inoculation.

Although both samples are differentiable trough simple eye-inspection, the absorbance intensity signal shown in Figure 6 is poor for quantification. This constraint is due to the screening by the turbid media itself which is a result of optical absorption in the visible spectrum by the bacterial cell components (membranes, organelles, DNA, proteins, etc.) (Gerhardt 1994; Koch 1970).

**Figure 7:**
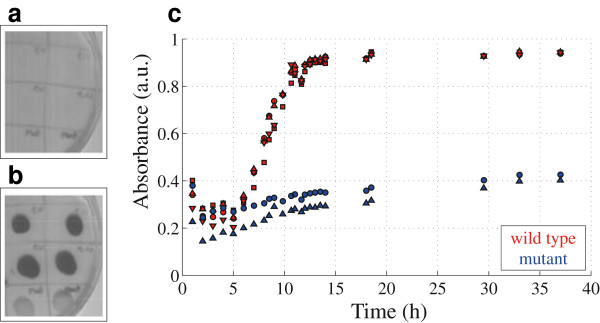
**Imaging at 580 nm of both** ***C. violaceum* wild-type strain (a) and** ***C. violaceum* CV026 strain (b).** Hyperspectral measurements in **(c)** show the absorbance intensity signal versus time for both strains.

With the aim to resolve the spectral differences between the strains associated with the pigment production, we use a PBHCs that allows to obtain spatio-spectral information without disturbing the sample. The experiment is developed by plating both strains in LB-agar at equal initial cell concentration, and following the methodology explained in the previous section. In particular, two spectral bands are selected and the subsequent temporal samples have information regarding the biological process. To obtain the signal, a band centered at 580 [nm] which corresponds to the wavelength of maximal absorption, is selected. In Figure 7c is shown the absorbance intensity signal versus time for both strains. Now the pigment absorbance signal is successfully retrieved and is clearly differentiable between the strains. The absorbance pigment intensity signal of the *C. violaceum* wild-type strain exhibits a bacterial growing-like curve behavior.

**Figure 8:**
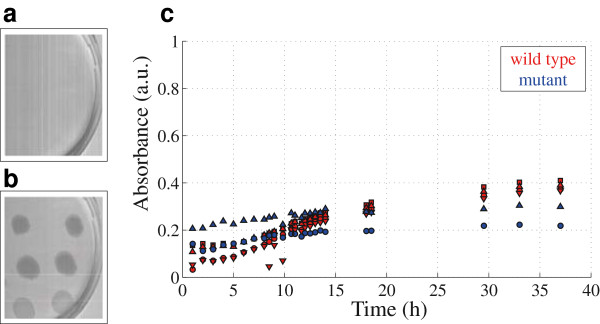
**Imaging at 764 [nm] of both** ***C. violaceum* wild-type strain (a) and** ***C. violaceum* CV026 strain (b).** Hyperspectral measurements in **(c)** show the absorbance intensity signal versus time for both strains.

In order to obtain quantitative information regarding the pigment production curves of Figure 7, some considerations need to be taken into account. The absorbance at 580 [nm] has simultaneous information of pigment and bacterial cell components. Thus, a band centered at 764 [nm] which corresponds to the minimal pigment absorption, is now selected. The absorbance intensity signal versus time for both strains at 764 [nm] can be seen in Figure 8.

**Figure 9:**
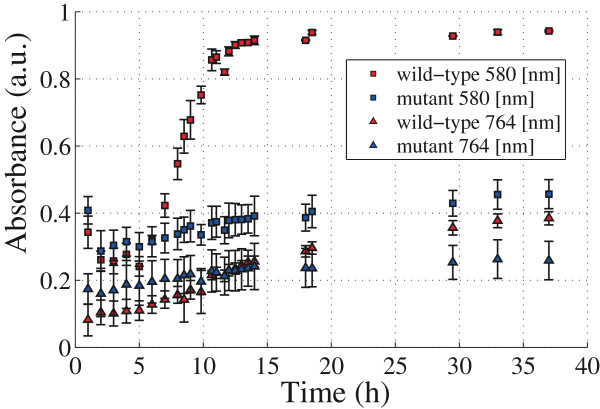
**Fitting of hyperspectral measurements of both *C. violaceum * wild-type strain and *C. violaceum * CV026 strain at 580 [nm] and 764 [nm].**

The information regarding pigment and cell components can be summarized in Figure 9. Data is fitted by using a non-linear least squares method on the exponential phase. Kinetic parameter of this fitting are shown in Table 1.

**Table 1:** Kinetic parameter of pigment production and bacterial growth

**Hyperspectral imaging measurement**^**a**^	**C.** ***violaceum***		**C.** ***violaceum***	
	**wild-type strain**		**CV026 strain**	
	**g(h)**^**c**^	**k (*h***^**-1**^**)**^**c**^	**g(h)**^**c**^	**k (*h***^**-1**^**)**^**c**^
580 [nm] (pigment production + bacterial growth)	5.28 ± 1.10	0.137 ± 0.029	29.77 ± 6.85	0.025 ± 0.006
764 [nm] (bacterial growth)	7.57 ± 1.14	0.094 ± 0.014	26.93 ± 7.31	0.028 ± 0.008
580 [nm]/764 [nm] (pigment production)	15.9	0.043	d	d
OD measurement ^b^	2.93 ± 0.65	0.249 ± 0.055	8.4 ± 1.23	0.08 ± 0.011

^a^measurements are made on solid medium with no agitation at RT.

^b^measurements are made on liquid medium with agitation at RT.

^c^The coefficients are estimated considering a confidence level of 95% that the real value is in that range.

^d^mutant strain does not show spectral response.

The grown parameters *g* and *k* for both strains at 764 [nm] are calculated. At this wavelength only information of bacterial growing is obtained. Although the wild-type strain grows faster than the mutant strain both have comparable velocities *k*. This is in agreement with the values found in literature and those obtained with the spectrophotometric measurements (see Table 1).

**Figure 10:**
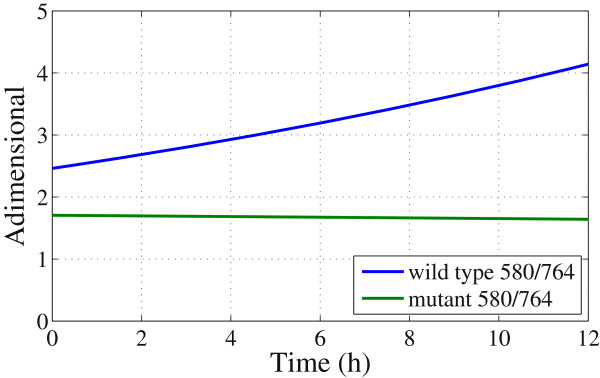
**Mathematical division of the fitting curves generated at 580 [nm] and 764 [nm] of both** ***C. violaceum* wild-type strain and** ***C. violaceum* CV026 strain.**

To quantify the violacein production, a division of functions is obtained. The foregoing is achieved between the curve regarding information of the bacterial pigment and bacterial growth (Figure 9)-squares and the curve containing information only of the bacterial growth (Figure 9)-triangles (Figure 10). Comparing the velocities of mutant strains at 580 [nm] and 764 [nm], noted that both values are equal, as expected, due to the lack of pigment production. *C. violaceum* CV026 strain are does not show spectral response in the spectral range analyzed.

## Discussion

The violacein production can be quantified using a spectrophotometer, after an extraction and purification process (Rettori and Duran 1998; Lu et al. 2009). This procedure fails to identify pigment remotely and not allows to follow the process in time. To follow the pigment production without destroying the bacterial cells we use a Hyperspectral Imaging System. This system provide spectral and spatial information of the *Chromobacterium violaceum* enabling us to obtain kinetic parameters of bacterial growth and pigment production.

The violacein production can be quantified at 580 [nm] by subtracting the contribution of the bacterial cell components at 764 [nm] which resulted in *k*_*pigment*_ = 0.043. Therefore, the results are in good agreement with the values calculated using optical density (OD) at 600 [nm] (see Table 1) and with those found in the literature (Corpe 1964).

In this work, a real time, non invasive and simple process to quantify bacterial pigment production trough HSI system is reported. The PBHC camera produces a hypercube with spatial and spectral information in function of time without destroying the sample. Hence we can analyze multiple samples with high wavelength resolution in a single experiment. Based on the process, the technique can be extensible to multiple colored samples at the same time. This optical technique could be useful for the analysis of complex samples such as bacterial biofilms, environmental samples or non-culture bacteria (Cortizo and de Mele 2000). This can be applied to quantify another biological pigments obtained from natural source without alter the environmental conditions. For example, studies can be done to quantify photosynthetic pigments, like chlorophylls or carotenoids, presents in some plants or bacteria. Also this technique can be used in the pigment determination in fruits or other exportation products. The advantage of this technique is the decomposition of the components of the biological system in a set of wavelengths each one associated to a specific spatio-temporal character. This allows to get new insights of a particular process in a complex media. Also, since we have detected the specific wavelengths that allows to characterize the bacterial pigment production and growing process, the next step is developing a filter-based imaging system with the aim to increases the temporal resolution of the measurements. That is, designing a monochromatic camera that includes a filter tuned at the wavelength of interest, discarding the scanning stage and acquiring the spatio-spectral image that exhibits greater variation in time (Nishino et al. 2013). Further, such devices do not require a scanning stage, which could increase the temporal resolution of our measurements.

## Competing interests

The authors declare that they have no competing interests.

## References

[B1] Ahmad WA Yusof N Nordin N Zakaria ZA Rezali MF Production and characterization of violacein by locally isolated *Chromobacterium violaceum* grown in agricultural wastes Appl Biochem Biotechnol 2012 4 1220 1234 doi:10.1007/s12010-012-9553-7 10.1007/s12010-012-9553-7 22278051

[B2] Arad SM Yaron A Natural pigments from red microalgae for use in foods and cosmetics Trends in Food Sci Technol 1992 4 0 92 97 doi:10.1016/0924-2244(92)90145-M

[B3] Borengasser M Hungate WS Watkins RL Hyperspectral remote sensing: principles and applications CRC Press, Series Remote Sensing Applications Series 2008 130 doi:10.1201/9781420012606.ch3

[B4] Cen H He Y Theory and application of near infrared reflectance spectroscopy in determination of food quality Trends in Food Sci Technol 2007 4 2 72 83 doi:10.1016/j.tifs.2006.09.003 10.1016/j.tifs.2006.09.003

[B5] Corpe WA Factors influencing growth and polysaccharide formation by strains of *Chromobacterium Violaceum* J Bacteriol 1964 4 5 1433 1441 14234803 10.1128/jb.88.5.1433-1441.1964 PMC277427

[B6] Cortizo M de Mele M Preliminary characterization of thin biofilms by optical microscopy BIOFOULING 2000 4 4 253 260 doi = 10.1080/08927010009386316 10.1080/08927010009386316

[B7] Duran N Menck C *Chromobacterium violaceum*: A review of pharmacological and industiral perspectives Critic Rev Microbiol 2001 4 3 201 222 doi:10.1080/20014091096747 10.1080/20014091096747 11596879

[B8] Duran N Justo GZ Ferreira CV Melo PS Cordi L Martins D Violacein: properties and biological activities Biotechnol Appl Biochem 2007 4 3 127 133 DOI:10.1042/BA20070115 10.1042/BA20070115 17927569

[B9] Gat N Imaging spectroscopy using tunable filters: a review. 2000 4 50 64 DOI:10.1117/12.381686

[B10] Gerhardt P Methods for general and molecular bacteriology 1994 Washington, DC: American Society for Microbiology 518 518

[B11] Jun W Kim M Lee K Millner P Chao K Assessment of bacterial biofilm on stainless steel by hyperspectral fluorescence imaging Sensing Instrum Food Qual Saf 2009 4 1 41 48 DOI:10.1007/s11694-009-9069-1 10.1007/s11694-009-9069-1

[B12] Kim TH Kong HJ Kim TH Shin JS Design and fabrication of a 900-1700 nm hyper-spectral imaging spectrometer Optics Commun 2010 4 3 355 361 DOI:10.1016/j.optcom.2009.10.022 10.1016/j.optcom.2009.10.022

[B13] Koch AL Turbidity measurements of bacterial cultures in some available commercial instruments Anal Biochemis 1970 4 1 252 259 DOI:10.1016/0003-2697(70)90174-0 10.1016/0003-2697(70)90174-0 4920662

[B14] Kong KW Khoo HE Prasad KN Ismail A Tan CP Rajab NF Revealing the power of the natural red pigment lycopene Molecules 2010 4 2 959 987 DOI:10.3390/molecules15020959 10.3390/molecules15020959 20335956 PMC6263198

[B15] Lazcka O Campo FJD Munoz FX Pathogen detection: A perspective of traditional methods and biosensors Biosensors and Bioelectronics 2007 4 7 1205 1217 DOI:10.1016/j.bios.2006.06.036 10.1016/j.bios.2006.06.036 16934970

[B16] Leon LL Miranda CC Souza AOD Durán N Antileishmanial activity of the violacein extracted from *Chromobacterium violaceum* J Antimicro Chemotherapy 2001 4 3 449 450 DOI:10.1093/jac/48.3.449 10.1093/jac/48.3.449 11533018

[B17] Lu Y Wang L Xue Y Zhang C Xing XH Lou K Zhang Z Li Y Zhang G Bi J Su Z Production of violet pigment by a newly isolated psychrotrophic bacterium from a glacier in Xinjiang, China Biochem Eng J 2009 4 2 135 141 10.1016/j.bej.2008.09.009

[B18] McClean KH Winson MK Fish L Taylor A Chhabra SR Camara M Daykin M Lamb JH Swift S Bycroft BW Stewart GSAB Williamsl P Quorum sensing and *Chromobacterium violaceum*: exploitation of violacein production and inhibition for the detection of n-acylhomoserine lactones Microbiol 1997 4 12 3703 3711 DOI:10.1099/00221287-143-12-3703 10.1099/00221287-143-12-3703 9421896

[B19] Nishino K Nakamura K Tsuta M Yoshimura M Sugiyama J Nakauchi S Optimization of excitation–emission band-pass filter for visualization of viable bacteria distribution on the surface of pork meat Opt Express 2013 4 10 12,579 12,591 DOI:10.1364/OE.21.012579. http://www.opticsexpress.org/abstract.cfm?URI=oe-21-10-12579 10.1364/OE.21.012579 23736477

[B20] Pisani M Zucco M Caricato V Egidi A Filtz J-R, Larquier B, Claudel P, et Favreau J. -O Hyperspectral imaging: a tool for biological measurements16th International Congress of Metrology 2013 14007 14007 doi =10.1051/metrology/201314007, http://dx.doi.org/10.1051/metrology/201314007

[B21] Plaza A Benediktsson JA Boardman JW Brazile J Bruzzone L Camps-Valls G Chanussot J Fauvel M Gamba P Gualtieri A Marconcini M Tilton JC Trianni G Recent advances in techniques for hyperspectral image processing Remote Sensing Environment 113 2009 4 0 S110 S122 DOI:10.1016/j.rse.2007.07.028

[B22] Rettori D Duran N Production, extraction and purification of violacein: an antibiotic pigment produced by *Chromobacterium violaceum* World J Microbiol Biotechnol 1998 4 5 685 688 10.1023/A:1008809504504

[B23] Sambrook J Fritsch E Maniatis T Molecular Cloning: a laboratory manual 1989 Cold Spring Harbor Laboratory Pr, N.Y., Cold Spring Harbor Laboratory

[B24] Sankaran S Ehsani R Etxeberria E Mid-infrared spectroscopy for detection of huanglongbing (greening) in citrus leaves Talanta 2010 4 2 574 581 DOI:10.1016/j.talanta.2010.10.008 10.1016/j.talanta.2010.10.008 21111177

[B25] Singh CB Jayas DS Paliwal J White ND Identification of insect-damaged wheat kernels using short-wave near-infrared hyperspectral and digital colour imaging Comput Electron Agric 2010 4 2 118 125 DOI:10.1016/j.compag.2010.06.001 10.1016/j.compag.2010.06.001

[B26] Sirimanne P Senevirathna M Premalal E Pitigala P Sivakumar V Tennakone K Utilization of natural pigment extracted from pomegranate fruits as sensitizer in solid-state solar cells J Photochemis Photobiol A: Chemis 2006 4 2-3 324 327 DOI:10.1016/j.jphotochem.2005.07.003 10.1016/j.jphotochem.2005.07.003

[B27] Siripatrawan U Makino Y Kawagoe Y Oshita S Rapid detection of *Escherichia coli* contamination in packaged fresh spinach using hyperspectral imaging Talanta 2011 4 1 276 281 DOI:10.1016/j.talanta.2011.03.061 10.1016/j.talanta.2011.03.061 21645699

[B28] Stahl W Sies H Bioactivity and protective effects of natural carotenoids Biochimica et Biophysica Acta (BBA) - Molecular Basis of Disease 2005 4 2 101 107 DOI:10.1016/j.bbadis.2004.12.006 10.1016/j.bbadis.2004.12.006 15949675

[B29] Steele DB Stowers MD Techniques for selection of industrially important microorganisms Ann Rev Microbiol 1991 4 1 89 106 DOI:10.1146/annurev.mi.45.100191.000513 10.1146/annurev.mi.45.100191.000513 1741626

[B30] Studer V Bobin J Chahid M Mousavi HS Candes E Dahan M Compressive fluorescence microscopy for biological and hyperspectral imaging Proc Nat Acad Sci 2012 4 26 E1679 E1687 DOI:10.1073/pnas.1119511109 10.1073/pnas.1119511109 22689950 PMC3387031

[B31] Velusamy V Arshak K Korostynska O Oliwa K Adley C An overview of foodborne pathogen detection: In the perspective of biosensors Biotechnol Adv 2010 4 2 232 254 DOI:10.1016/j.biotechadv.2009.12.004 10.1016/j.biotechadv.2009.12.004 20006978

[B32] Vo-Dinh T A hyperspectral imaging system for in vivo optical diagnostics Eng Med Biol Mag, IEEE 2004 4 5 40 49 DOI:10.1109/MEMB.2004.1360407 10.1109/MEMB.2004.1360407 15565798

[B33] Zavattini G Vecchi S Leahy R Smith D Cherry S A hyperspectral fluorescence imaging system for biological applications Nuclear Science Symposium Conference Record 2003 2003 IEEE 942 946, Vol.2 DOI:10.1109/NSSMIC.2003.1351850

